# Gastric metastasis and peritoneal carcinosis revealing primary breast cancer: an unusual presentation

**DOI:** 10.2144/fsoa-2023-0182

**Published:** 2024-06-17

**Authors:** Shema Ayadi, Souhir Monastiri, Amine Ben Safta, Mehdi Hammami, Imen Samaali, Mehdi Kammoun, Ahlem Blel, Raoudha Aloui, Yosra Zaimi, Leila Mouelhi

**Affiliations:** 1 Gastroenterology Department, Charles Nicolle university hospital, Tunis, Tunisia; 2 Surgery B Department, Charles Nicolle university hospital, Tunis, Tunisia; 3 Pathology Department, Charles Nicolle university hospital, Tunis, Tunisia; 4 Faculty of Medicine of Tunis, University of Tunis El Manar, Tunis, Tunisia

**Keywords:** breast cancer, GI tract, lobular breast carcinoma, metastases, peritoneal carcinosis

## Abstract

Breast cancer is the most frequent cancer among women. Gastrointestinal tract metastases are uncommon and might be misidentified as primary carcinoma.

A noteworthy case-study involved 53-year-old-woman complaining from epigastric pain, ascites and overall health decline. Initial investigations were inconclusive, prompting laparoscopic peritoneal biopsies which revealed independent cell proliferation. Subsequently, a second look upper digestive endoscopy showed multiple gastric ulcerations suggestive of gastric carcinoma. Histologic examination confirmed independent cell proliferation with estrogen receptors expression, a characteristic feature of breast carcinoma. Further investigations led to bilateral invasive lobular breast carcinoma diagnosis. Epirubicin cycophosphamide was prescribed after progression under letrozole ribocilib therapy.

This case aims to raise awareness among clinicians about the importance of ruling out breast cancer in patients with peritoneal carcinosis and paying attention to digestive symptoms in breast cancer patients with careful gastric endoscopic examination to avoid misdiagnosis.

## Abstract (plain language summary)

Gastrointestinal metastases and peritoneal carcinosis represents rare metastatic sites of breast cancer with unfavorable prognosis.

Invasive lobular carcinoma is the most frequent histologic type involved in gastrointestinal metastases.

Digestive clinical symptoms are not specific and indicates upper-digestive endoscopy with careful gastric mucosa examination and biopsies to avoid misdiagnosis.

Independent cells proliferation with linear cords distribution within the gastric mucosa should evoke the diagnosis of gastric metastasis of lobular carcinoma.

Immunohistochemistry is mandatory to distinguish between gastric metastasis of breast carcinoma and primitive gastric carcinoma. Typical profile of gastric metastasis is ER positive, PR positive, CK7 negative and CK20 negative.

Breast carcinoma has been identified as the predominant cancer among women, constituting approximately 29% of all recently diagnosed cancers in young women [1]. Over time, these carcinomas have shown a tendency to spread to various parts of the body, including the lymph nodes, lungs, bones and liver. Notably, metastasis to the gastrointestinal (GI) tract is a rare occurrence, accounting for less than 1% of cases. When breast cancer does metastasize to the stomach, it typically associates with advanced stages of the disease or emerges several years after the initial treatment.

Studies over the past years have consistently reported divergent metastatic tendencies between lobular and ductal carcinoma. There is a notable inclination for lobular carcinoma to metastasize to the GI tract, gynecological organs and the peritoneum, while ductal carcinoma more frequently exhibits relapses in the liver, lungs and brain.

Gastric metastases from breast cancer have been observed to imitate primary gastric tumors in their clinical presentation as well as their endoscopic findings. When a gastric tumor is identified in a patient with a history of breast cancer, it was previously inclined to be considered a primary gastric lesion. However, the passage of time has emphasized the importance of thorough investigation to rule out the possibility of metastasis from breast cancer. Interestingly, there have been no reported instances over the years of gastric metastases or peritoneal carcinosis revealing the presence of primary breast carcinoma. In this report, we delve into a unique case where gastric and peritoneal metastases, observed over time, provided crucial clues leading to the diagnosis of a primary invasive lobular breast carcinoma.

## Case report

A 53-year-old menopausal female patient, devoid of any family history of neoplasia and lacking personal pathological antecedents, was admitted to the gastroenterology department due to abdominal distention, epigastric pain and an overall health decline that had been progressing for the past 3 months. Upon clinical examination, ascites was evident with no palpable abdominal mass or adenopathy. There were no features indicative of right cardiac failure. Laboratory tests revealed a C-reactive protein level of 18 mg/l without leukocytosis, inflammatory anemia with a hemoglobin level of 11.1 g/dl, and an elevation in serum alkaline phosphatase levels to 1.8-times the normal range (284 UI/l). Biochemical and cytologic analysis of ascites fluid demonstrated a protein rate of 40 g/l and a leukocyte rate of 200 E/mm^3^, suggestive of peritoneal carcinosis.

**Figure 1.**
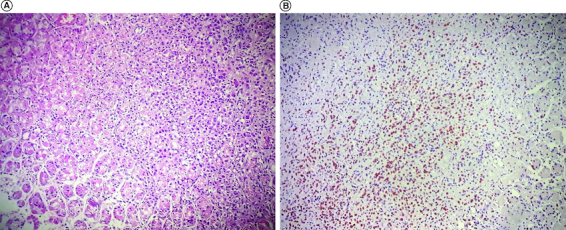
Pathologic findings of gastric biopsies. **(A)** Tumoral proliferation of independent cells in gastric mucosa (× 200). **(B)** Immunostaining of tumor cells for estrogen receptors (× 100).

Several investigations to search for primary metastatic site was conducted. As the patient experienced epigastric pain, upper and lower digestive endoscopies were first performed and yielded inconclusive results. Subsequently, a thoraco-abdominal and pelvic CT scans was performed indicating abundant ascites and thickening of the cervix wall. To explore the cervix wall thickening, a complementary gynecologic examination and pelvic MRI ruled out pelvic neoplasia. Primary metastatic site remained unknown indicating a diagnostic laparoscopy. Per-operative findings showed abundant ascites, a nodular appearance of the greater omentum, small intestine, colon and a micronodular aspect on the parietal peritoneum consistent with peritoneal carcinosis. Perioperative biopsy examination demonstrated the proliferation of independent cells. Considering the epigastric pain experienced by the patient and given the patholologic findings, a second upper digestive endoscopy was conducted. It revealed multiple fundic erosions and superficial ulcerations. Pathologic examination of gastric biopsies indicated the presence of independent uniform cells arranged in a single-file line, highly suggestive of breast carcinoma metastasis (Figure 1).

Further investigation confirmed the diagnosis of breast lobular carcinoma through positive screening for estrogen receptor expression.

Subsequent breast ultrasounds and mammography exhibited multiple bilateral nodules classified as ACR4 bilaterally. Pathologic examination of breast nodules confirmed infiltrating lobular carcinoma with a negative expression of HER2 receptors. A second staging thoracoabdominal and pelvic CT scan revealed abundant ascites and multiple bone metastases.

Based on these findings, the patient was diagnosed with invasive lobular breast cancer with gastric, peritoneal and bone metastases. Consequently, she underwent letrozole–ribocilib therapy as first treatment. Epirubicin–cycophosphamide cycles were then prescribed, after rapid progression, within 5 months, according to RECIST criteria.

## Discussion

Breast cancer is the most prevalent cancer among women and the primary cause of cancer-related deaths in those aged 20 to 59. It represents 29% of all newly diagnosed cancers in women and is responsible for 14% of their cancer-related deaths [1]. The prevalence of metastatic gastric cancer from breast carcinoma has been reported to be as low as 0.1–0.5% [2,3]. Nevertheless, in a comprehensive study examining the autopsies of 1010 patients diagnosed with cancer, researchers found gastric metastases in only 17 patients. This notably low occurrence rate, less than 1.7%, underscores the rarity of this particular metastatic manifestation [4,5]. This suggests that the prevalence of metastatic gastric cancer from breast carcinoma has been underestimated with several cases being probably not diagnosed. So that, more attention should be drawn to minor digestive signs to avoid missing the diagnosis of such metastases. Gastric metastases typically occurs several years after the initial diagnosis of primary breast cancer and is associated, in 90–94% of cases, with other concomitant metastases [6,7]. According to Hong *et al.*‘s study, the stomach was identified as the primary site of metastatic lesions in 6 out of 11 patients (54.5%) diagnosed with *de novo* stage I to III cancer. Among these six patients, the median time to gastric metastasis was 77.5 months. In the remaining five patients, the median duration between the onset of initial distant metastases and gastric involvement was 42 months, with variations in the sites of initial metastatic lesions [8]. Our case, on the other hand, presented digestive symptoms along with peritoneal carcinosis, leading us to diagnose breast cancer.

To our knowledge; there have been no reports in the literature of peritoneal carcinosis or gastric lesions diagnosed before the primary breast carcinoma. This is the first case of peritoneal carcinosis and gastric metastases revealing lobular breast carcinoma which highlights the originality of this study. This should, firstly, raise awareness among clinicians about the imperative need to thoroughly investigate and rule out breast cancer metastases in patients presenting with peritoneal carcinosis and secondly to pay more attention to clinical symptoms of gastric metastases in patients with already known breast cancer.

In fact, clinical presentation of gastric metastases can be similar to that of primary gastric cancer, making it challenging to differentiate between the two conditions. Common symptoms include dyspepsia, anorexia, epigastric pain, early satiety, vomiting and bleeding [9,10]. These symptoms may be also misdiagnosed as chemotherapy toxicity which can also explain the higher prevalence of gastric metastases in above cited autopsy series. Moreover, this underlines the importance to perform endoscopic investigation in breast cancer patients experiencing digestive symptoms. The most frequently observed pattern of breast cancer gastric metastasis mimics linitis plastica (73%) [6,9,10]. Other endoscopic features were described although they were less common: multiple nodules of varying sizes and numbers, nonulcerative masses, submucosal tumor masses with elevation and tip ulceration and external compression of the stomach [7,10]. In addition, in other previous reports, gastric lesions were described as either polypoid masses, volcano-like ulcers or isolated erosion in the gastric corpus and predominantly affected the lower third of the stomach, including the antrum and pylorus [9,11]. In our case, first endoscopic investigation was inconclusive and the second one showed multiple gastric ulcerations. This can be explicated by a rapid progression of the gastric lesions. The possibility of the presence of minor lesions on the first digestive endoscopy cannot be ruled out which highlights the need of a careful examination of the gastric mucosa in the context of a patient with breast carcinoma and epigastric pain. In addition, Al Moubarek *et al.* discovered that gastric metastatic lesions were located in the fundus in 15 cases (43%), in the antrum in 15 cases (43%) and in both locations in 5 cases (14%) [12]. It is important to note that gastric metastasis occurs preferentially in lobular breast carcinoma. In fact, different histological types of primary breast cancer are associated with different sites of metastasis. Compared with invasive ductal cancer, invasive lobular carcinoma is more likely to have distant metastases, particularly in the GI tract, peritoneum, ovaries and uterus [13,14]. In addition, according to Taal et al., 83% of patients with gastric metastasis had invasive lobular carcinoma [6], which is consistent with our findings. However, metastases stemming from invasive lobular carcinoma typically display a distinct pattern of widespread infiltration within the stomach, although, as observed in our particular instance, this presentation can be variable. Due to the atypical clinical imaging and pathological findings, immunohistochemistry plays a crucial role in the diagnosis of this entity. The bulk of metastatic breast carcinomas demonstrated positivity for ER and PR receptors while being negative for CK20. Conversely, primary stomach cancer usually exhibits positivity for CK7 and CK20, along with negativity for ER and mammaglobin receptors. Despite this, HER2, a well-established contributor to the carcinogenic pathway, undergoes aberrations in several solid tumor types such as breast and stomach cancer implying that it might not serve as a suitable diagnostic indicator for identifying gastric metastasis originating from breast cancer [15]. ER and PR, commonly employed as prognostic markers in breast cancer, can yield positive results in 32 and 12% of gastric cancer patients, respectively. However, they do not serve as effective tools to distinguish between primary gastric cancer and gastric metastasis originating from breast cancer. Additionally, HER2, routinely assessed in primary gastric cancer cases, lacks utility due to its low occurrence in lobular carcinomas (5.9% of cases). Furthermore, the intratumor heterogeneity of HER2 expression is notably higher in primary gastric cancer compared with breast cancer. Gastrointestinal metastasis of breast carcinoma indicates palliative treatment chemotherapy, hormone therapy and targeted therapy [15]. The selection of systemic treatment relies on various factors, including age, current symptoms, performance status, ER status and treatment history [16]. In Taal's study, partial remission was observed in 46% of cases and 52% of patients achieved stabilization, while 20% experienced regression [17]. Specific-localization treatment may be needed in rare cases, Including palliative surgical resection [18], and endoscopic interventions similar to primary gastric cancer. In fact, gastric outlet obstruction, a common complication, can be addressed using endoluminal stents. For controlling bleeding, both endoscopic and endovascular therapies can be employed [19,20]. Surgical resection is typically limited in its roles since gastric metastasis indicates a systemic disease [21], and studies showed that palliative surgery did not significantly impact overall survival [18]. Therefore, surgical interventions should be reserved for palliative bypass procedures when less invasive measures fail to alleviate gastric outlet obstruction in selected patients. The prognosis of metastatic breast cancer involving the GI tract is generally unfavorable [9]. The median survival time after diagnosis of gastric metastasis is 10 months [17], with a three-year survival rate of 79.1% [8]. This is primarily due to the fact that the GI tract is not a common site of metastasis in breast cancer, and when gastric metastasis does occur, it often indicates an advanced stage of the disease. However, it is worth noting that in cases where breast carcinoma extension is limited to oligometastatic disease, the prognosis tends to be relatively better compared with those with widespread metastasis [9].

## Conclusion

Gastrointestinal metastases of breast carcinoma are rare and occurred more frequently in patients with invasive lobular breast carcinoma. This must raise awareness among clinicians about importance of excluding breast malignancies during etiologic investigations of peritoneal carcinosis and to pay more attention to digestive symptoms in breast cancer patients to avoid misdiagnosis of gastric metastases and adapt treatment. Pathologists should be also mindful of the possibility of metastatic lobular carcinoma instead gastric independent cells proliferation.
